# Navigating HIV citizenship: identities, risks and biological citizenship in the treatment as prevention era

**DOI:** 10.1080/13698575.2019.1572869

**Published:** 2019-01-31

**Authors:** Ingrid Young, Mark Davis, Paul Flowers, Lisa M. McDaid

**Affiliations:** a Centre for Biomedicine, Self and Society, Usher Institute, University of Edinburgh, Edinburgh, UK; b School of Social Sciences, Monash University, Melbourne; c MRC/CSO Social and Public Health Sciences Unit, University of Glasgow, Glasgow, UK

**Keywords:** HIV, treatment as prevention, biological citizenship, people living with HIV, gay men, African communities

## Abstract

The use of HIV Treatment as Prevention (TasP) has radically changed our understandings of HIV risk and revolutionised global HIV prevention policy to focus on the use of pharmaceuticals. Yet, there has been little engagement with the very people expected to comply with a daily pharmaceutical regime. We employ the concept of HIV citizenship to explore responses by people living with HIV in the UK to TasP. We consider how a treatment-based public health strategy has the potential to reshape identities, self-governance and forms of citizenship, domains which play a critical role not only in compliance with new TasP policies, but in how HIV prevention, serodiscordant relationships and (sexual) health are negotiated and enacted. Our findings disrupt the biomedical narrative which claims an end to HIV through scaling up access to treatment. Responses to TasP were framed through shifting negotiations of identity, linked to biomarkers, cure and managing treatment. Toxicity of drugs – and bodies – were seen as something to manage and linked to the shifting possibilities in serodiscordant environments. Finally, a sense of being healthy and responsible, including appropriate use of resources, meant conflicting relationships with if and when to start treatment. Our research highlights how HIV citizenship in the TasP era is negotiated and influenced by intersectional experiences of community, health systems, illness and treatment. Our findings show that the complexities of HIV citizenship and ongoing inequalities, and their biopolitical implications, will intimately shape the implementation and sustainability of TasP.

## Introduction

In July 2016, the social media campaign #U = U (Undetectable = Untransmittable) was launched by the Prevention Access Campaign, a collection of clinicians, researchers, community and HIV organisations (Prevention Access Campaign, 2016). The aim was to reduce HIV stigma by highlighting Treatment as Prevention (TasP). That is, HIV treatment in people living with HIV can not only manage the HIV in their bodies, but also *prevent* onward transmission. While most HIV and community organisations actively endorsed this message, the Global Network of People Living with HIV (GNP+) – one of the biggest organisations of people living with HIV – were initially highly critical of the campaign. They argued that promoting this message ‘as a stand-alone brings uncomfortable reminders of prevention with positives’ programme that put the responsibility of prevention solely on people living with HIV’ (GNP+, 2017, p. 1). This response was not only highly criticised by most other HIV and community organisations, but it was also not in keeping with global rhetoric in HIV prevention which had embraced TasP as a prevention strategy.

The initial suggestion that an undetectable viral load would reduce transmission in the 2008 Swiss Statement (Bernard, 2008) was widely rejected on the grounds that it was based on an observational study, not a clinical trial. However, results from clinical trial HPTN052 in 2011 and its 2015 follow-up study showed that TasP could reduce HIV transmission by 93% and consolidated the idea within the medical community that treatment *was* prevention (Cohen, Chen, & McCauley, et al., 2015). Although TasP began to be seen as an effective community-level prevention strategy as a result of HPTN052, there was some debate about the potential burden on people living with HIV starting treatment for prevention, rather than for their own care (Sabin, Cooper, Collins, & Schechter, 2013). In 2015, the START trial reported interim findings that those starting treatment immediately after diagnosis had a significantly lower risk of illness and death compared to those who delayed treatment (INSIGHT START Study Group, 2015). With significant, gold-standard evidence that the use of anti-retrovirals (ARVs) in individuals living with HIV is beneficial for both their health and HIV prevention, any controversy around the biomedical TasP narrative, and related public health policies, disappeared. The way forward was to implement TasP policy globally (Beyrer et al., 2015).

Although access to treatment has long been framed as a right (Robins & Von Lieres, 2004), ensuring people living with HIV secure this right has become more urgent in the TasP era. Yet, consultation with those most affected by HIV in relation to TasP has been slow to emerge. Some research with communities of people living with HIV has been published (Carter et al., 2015; Grace et al., 2015; Keogh, 2017; Newman et al., 2015a, 2015b; Persson, 2013; 2015; Young, Flowers, & McDaid, 2016). Broadly speaking, this research highlights ambivalence towards TasP as a public health strategy, anxieties and difficulties around starting and accessing treatment, and mixed levels of knowledge and engagement with the new science of HIV. As TasP is rolled out globally as a core ingredient of HIV prevention policy, what are the implications of this disconnect between policy, implementation and the experiences of those diagnosed with HIV – those tasked with the responsibility to comply with a daily ARV regime? In this article we explore responses to TasP among people living with HIV in the UK. We draw on qualitative research with participants living with HIV who have mixed experiences of treatment. We explore the implications for an individual with a chronic illness invited to start treatment not only for their own health, but to prevent transmission to others. As such, we consider how a treatment-based public health strategy has the potential to reshape identities, self-governance and forms of citizenship, domains which play a critical role not only in compliance with new TasP policies, but in how HIV prevention, serodiscordant relationships and (sexual) health are negotiated and enacted.

## Citizenship and public health

TasP has been described as part of the wider biomedicalisation of HIV prevention and care (Keogh, 2017; Young et al., 2016). One of the key elements of biomedicalisation is biological citizenship (Clarke, Mamo, Fosket, & Shim, 2010). Petryna’s (2002) description of biological citizenship considers how rights to citizenship and health were negotiated within the aftermath of the Chernobyl disaster in the Ukraine. Petryna links the embodied biotechnological practices of managing radiation poisoning and gaining access to healthcare and treatment to civic and welfare rights. Building on Petryna’s work, Rose (2007) argues that developments in biotechnology represent a significant shift in the way we understand and govern bodies and their possibilities. Biological citizenship has implications for how we understand and manage life itself. As life is being shaped and reshaped at the molecular level as a result of new biotechnologies, Rose argues that the ethical relationship to our bodies has also changed and that our ‘corporeality,’ not just conduct, has become subject to Foucault’s ‘technologies of the self’ (Rose, 2007). In other words, the imperative of health is not only to engage in appropriate, healthy activities but to strive to *be* healthy. Where Peterson and Lupton (Petersen & Lupton, 1996) describe healthy citizenship and the imperative to follow a healthy lifestyle, biological citizenship goes a step further, where citizens are encouraged to be healthy not only through the physical management of bodies (e.g. exercise), but also by using biotechnologies for diagnosis, treatment and ongoing (or anticipatory) monitoring. Citizenship in the era of biomedicalisation is governed through both rights and responsibilities: the rights to biotechnologies, treatment and care and the responsibility for the health and well-being of oneself and others.

In the TasP era, HIV treatment has shifted from a regime of self-care, to an enhanced regime of self-monitoring to ensure the future health of others (Keogh, 2017; Squire, 2013). The notion of biological citizenship is especially pertinent in the context of TasP and HIV treatment more broadly. Building on Rose’s work, Nguyen’s concept of therapeutic citizenship describes how subjects are formed through an assemblage of HIV institutions that make up the global AIDS industry. This citizenship encompasses activism, peer support and counselling techniques, where citizens learn to tell a story and are triaged into treatment regimes in resource-scarce settings as valuable members of emerging HIV communities (Nguyen, 2010). Russell, Namukwaya, Zalwango, and Seeley (2016) in their work on HIV treatment in Uganda suggest that therapeutic citizenship is characterised by self-efficacy, a commitment to self-management and a desire to take ownership of one’s health (Russell et al., 2016). Here we see not only a sense of self-care and access to treatment, but a responsibility towards others in the form of self-monitoring to maintain undetectable viral loads. These changes signal increasing complexity in ways of living with and/or embodying HIV, HIV identities and enactments of HIV citizenship.

Paparini and Rhodes (2016) reviewed research which engaged with ideas of biological and therapeutic citizenship in their accounts of HIV and its treatment (Paparini & Rhodes, 2016). With most research focused on HIV experiences of treatment in the Global South, the authors suggest that the concept of citizenship may be limited in the context of alternative forms of knowledge and expertise, material inequalities and poverty, and a virtual absence of biotechnologies and health systems. However, we are interested in citizenship as it governs self-management (bodily and otherwise) and ascribes identity to someone in the context of a wider, biosocial collective. How does being a part of a ‘community’ of people living with HIV affect one’s self-care and care of others within that ‘community’ in the context of TasP? While our research focuses on experiences of HIV within the UK, it does so by looking at communities (epidemiologically determined, but socially imagined) and recognising the heterogeneity of these groups tied together through an enhanced risk and/or experiences of HIV. In this paper, we seek to understand HIV citizenship and how it is negotiated within a significant epistemic shift of TasP. Here, we understand HIV citizenship to be a *form* of biological and/or therapeutic citizenship, but which is specific to the experiences of people living with the virus. We pay close attention to what it means to live with or embody HIV, if and where this elicits alignments with others who are also living with HIV and how HIV-specific biosocial identities and practices are shaped within the TasP era. Paparini and Rhodes call for further research on emerging forms of patient citizenship linked to care and pharmaceutical demands, especially in light of increased competition for public funding. They suggest it will be important to analyse how biological citizens may be increasingly made individually responsible for more than their own health in the context of TasP. Our article responds to this by exploring HIV citizenship to understand experiences of living with HIV in the UK at the start of the TasP era. How does the transformation of HIV affect identity and prevention practices for people living with HIV already on treatment and for those who are now faced with the possibility of treatment in this new era? What are the biopolitical implications of the pharmaceuticalisation of HIV prevention and the subsequent demands on people living with HIV to participate as a matter of their lives? If citizenship is about assuming rights, claiming an identity and negotiating responsibilities, how does the imperative to take treatment *for* prevention shape, and how is it shaped by, the experiences of HIV citizenship? Our research has important implications not only for the implementation of pharmaceutically based HIV prevention policies, but also for the voices of people living with HIV. We explore how the complexities of HIV citizenship and ongoing inequalities shape the implementation and sustainability of TasP.

## Research methods

We draw on qualitative research conducted with communities most affected by HIV in Scotland, where gay and bisexual men, and migrant African women and men living in the UK are most affected. Rates of HIV amongst these communities have continued to increase until quite recently (Kirwan, Chau, Brown, Gill, & Delphech et al., 2016). The Scottish health system provides access to HIV treatment and care to anyone diagnosed with HIV at no cost at the point of delivery, including treatment. The policy in relation to TasP at the time this research was conducted was that treatment initiation for the purposes of HIV prevention was available on a case by case basis (BHIVA, 2014). In practice, only about 10% of people diagnosed with HIV in the UK are not currently taking treatment. In September 2015, new HIV treatment guidelines developed by the British HIV Association (BHIVA) recommend treatment immediately to all people diagnosed with HIV for the purposes of prevention, effectively pursuing a TasP policy. (Churchill, Waters, Ahmed, Angus, & Boffito et al., 2015) How this will take place, what effect this will have on numbers of individuals living with HIV who are on treatment or even how TasP will be introduced to patients – either as good for their own health and/or as a means of reducing the possibility of infection – remains uncharted.

This research was conducted in 2013 with communities affected by HIV to inform the potential and/or impending implementation of TasP and Pre-exposure prophylaxis (PrEP), the provision of ARVs to HIV-negative individuals to prevent the acquisition of HIV. This research, part of the wider project *HIV and the Biomedical*, sought to understand how significant changes in scientific understandings of HIV risk as a result of PrEP and TasP would be understood and received by communities affected by HIV. Participants of any HIV-diagnosis were recruited from two communities most affected by HIV in Scotland: gay and bisexual men and African women and men living in Scotland. We undertook 34 interviews in total, with 20 gay and bisexual men and 14 African participants. Working with partners from clinical and community organisations who work in and around HIV and sexual health, we recruited participants using flyers and posters targeted at either gay and bisexual men, or African communities, which were distributed through clinical (HIV and/or sexual health clinics), community and/or culturally specific, non-sexual health venues. Community partners also advertised the research in relevant digital settings, such as Facebook groups.

We used semi-structured interviews that lasted between 60–120 min. Interviews took place in community organisation offices, university offices or in participants’ homes, and were conducted by IY and PF. Participants received £20 vouchers for their participation. Interviews were digitally recorded, and transcribed. The first part of the interview focused on personal experiences of and perspective on HIV, sexual health risk management practices and use of sexual health technologies. To prompt discussion, we presented participants with a list of sexual health technologies (e.g. condoms, contraceptive devices, self-testing technologies, etc.). The second part of the interview focused on the acceptability of PrEP and TasP, exploring awareness, potential use, concerns and potential use with existing risk management strategies. For participants living with HIV, we introduced the concept of TasP as a prevention strategy where ARVs were initiated for the purposes of preventing transmission to sexual partners. For further information on methods, include topic guides and interview aids, see Young, Flowers, and McDaid (2015). Our research received ethical approval from University of Glasgow College of Social Sciences Ethics Committee (CSS2012/0264). For this article, we undertook a thematic analysis on a sub-set of in-depth interviews with 17 participants living with HIV: 10 with gay and bisexual men; and six with African women and one with an African man. Participants ranged from 24 to 60 years old, and lived across four Scottish regions: Glasgow, Edinburgh, Lanarkshire and Grampian. Of these participants, only three were not on treatment at the time of interview, with one reporting treatment during pregnancy. Length of time living with HIV also varied considerably amongst participants, with the earliest diagnosis in the late 1980s to the most recent diagnosis less than a year before the interview took place. Interview transcripts were uploaded to NVivo10, and a preliminary inductive thematic analysis was undertaken primarily by IY, by reading and re-reading the interview transcripts to identify themes. Through presentation of an initial set of themes, and subsequent discussions between the IY, MD and PF, a set of final themes were agreed. Authors drew on an interpretive approach to identify and agree final themes. Rigour throughout the analysis was achieved through an iterative process of discussion and revisions of findings (Mason, 2002; Silverman, 2000).

## Findings: treatment as prevention discourse on HIV citizenship

Our findings show responses to TasP were strongly linked to experiences with HIV treatment and understandings of how to live with and manage HIV. We present three themes which emerged from discussions and which affect understandings and responses to TasP in the context of HIV citizenship: pharmaceutical positioning; toxicity and the *responsible* use of resources.

## Pharmaceutical positioning

Consistent with research on identity and ARVs (Flowers, 2010), we found the capacity of ARVs to produce undetectable viral loads impinged upon how participants talked about their embodied experiences of living with HIV, and how this related to understandings and, relatedly, expressions of their biosocial HIV identities. Most participants who were taking ARVs described themselves, unprompted, as *undetectable* with many providing their most recent CD4 counts. This highlights the ways in which nearly all participants on treatment sought to present themselves as adherent to their drug regimes. It also clearly speaks to the action of treatment upon the HIV virus within the individual embodied subject. However, through the rhetoric of TasP and the ‘other-oriented’ implications of biomedicalisation, it also addresses the ‘HIV-positive’ body as an object sequestered for public health. One participant described the monitoring of viral loads in the lead up to treatment and, then, the dramatic and welcome impact of ARV on his viral load:

I was still going every few months or so and I was constantly getting …“no, your CD4 count’s really good and your viral loads always low” and it seemed to really tick away like that for ages so… I probably did in the back of my head, I would be thinking ‘someday I’m probably going to go on meds, you know? But it sounds like it’s going to be a very long, long time away.’ But it was all very sudden, when it did happen it was quite quick and… it’s sad to say, what, 99% of me is glad I’m on meds as well ‘cause I know it keeps me in the right place. I’m undetectable and, you know, I don’t have any ill-effects so I’d rather be like that than, I guess, fluctuating up and down. (Gay man, 40s, on ARVs)

The dramatic transition highlighted in this account connects HIV citizenship with the historical and biographical shifts and changes in HIV treatment. The interviewee hints at the loss of his identity as someone who did not require treatment, as healthily living with HIV who then transitioned to be someone ‘in the right place,’ with an undetectable viral load. The account seemed to mark the termination of a liminal identity of ‘not unwell but in need of treatment’ and figured in terms of a notional, ‘successful’ person with HIV. ARV bio-markers such as *undetectable* appeared to serve as the identity labels of ‘good patients,’ who comply with their ARV prescription and therefore ‘successfully’ manage their HIV infection. However, we also found considerable diversity in engagements with the TasP culture of identities and biomarkers. For another man, the presence or absence of HIV infection remained significant to his sense of self with HIV:

…until someone’s says like “your viral load has been non-detectable for the last six months, year, two years, whatever, we feel you can come off medication,” as long as you’re on medication, you’re suppressing something… even though it’s undetectable and you may be in a safer bit, and the point of actually infecting someone may be never, but you’re still on the medication so it’s not primarily a cure…it’s just suppressing, maintaining… ‘cause if you stop the medication, it’s gonnae come back. (Gay man, 50s, on ARVs)

This participant highlights the durability of his HIV identity despite his ‘good’ HIV citizenship. This approach may point to generation and membership of gay community, encompassing the long-established HIV prevention rationality for gay men (Flowers, 2001) and related discourse on the dangers of treatment optimism (Elford, Sherr, Adam, Crawford, & Kippax et al., 2003). This respondent is adamant about his HIV status, regardless of an undetectable viral load. He is in this sense always ‘HIV-positive’ and, therefore, always poses a risk to others. His acceptance of TasP is provisional. It does not erase pre-existing constructions of identity and responsibility to others. Indeed, here we see that his HIV identity plays an integral role in negotiations of responsibility, especially in relation to HIV prevention (Young et al., 2015). Currently and because of the effectiveness of ARV’s in the TasP era, HIV bioscience is focused on achieving a cure, which also includes the notion of a functional cure where viral loads can be suppressed without the long-term use of ARVs (Rennie, Siedner, Tucker, & Moodley, 2015). How the shift to cure might impinge on the HIV identity of this man and others like him, is an open question and helps to underline the fluid character of the TasP discursive environment.

Participants in our study worked hard to enact forms of good HIV citizenship. However, doing so in social interaction could be challenging. Generational differences in knowledge of TasP, as well as a divide associated with those taking ARVs due to their serostatus, played a critical role in the expression of this HIV citizenship:

I mean I bumped into an eighteen year old…[and] through nothing I said brought up sort of HIV with some level of awareness but you know, “not that I’ve got HIV or anything or what is it, AIDS, I haven’t got the AIDS.” You know, that’s still what people are talking about and when you start to say even in similar term- “well, HIV’s not AIDS babe.” “Oh how do you know, have you got it?” … sort of thing. It kinda puts you into… and when I could start saying all about CD4 counts and viral loads and medications and it’s a chronic illness, you know, it’s no longer considered fatal blah blah, it does, I think it starts to expose you a little. (Gay man, 30s, on ARVs)

Previous research has demonstrated that gay men living with HIV have more nuanced and effective ARV knowledge than do HIV-negative gay men (Rosengarten, Race, & Kippax, 2000). Our research indicates the persistence of divisions among gay men according to HIV serostatus. The respondent describes how discussing HIV and viral loads would expose him in social life, highlighting how the TasP environment is influenced by enduring fears and prejudices regarding HIV. Counter to the public health celebrations of TasP, the individual costs of enacting HIV citizenship are clearly outlined. The participant goes on to say how he explained his HIV knowledge because he was a volunteer for an HIV charity. This suggests that knowledge of HIV, and especially knowledge of treatment and its role in prevention, is delimited by serostatus and that this boundary is enforced by the stigma.

In contrast and reflecting a strikingly full-fledged endorsement of the potential of TasP, another participant described themselves as ‘HIV-negative:’

I’m HIV-negative. Actually, I was diagnosed in 2008 and I was found positive and immediately the doctors had a discussion with me, ‘cause I think they found my CD4 count was really low, whether to go on medication or not. So my decision was to actually go on medication and going back, three months after that … then I was negative. And from 2008, it’s kind of like it’s been an amazing journey for me with the medication they put me on. I’ve been completely negative, the viral load has been undetectable, yeah… I would say I’m living with HIV which is being controlled by the medication. So, the viral load, the results are always negative, yeah… Yeah always undetectable. It’s been negative throughout. (African woman, 30s, on ARVs)

This slippage between undetectable and negative appeared to be an intentional effect of this participant’s discourse on her health and formulation of identity. It is as though her ‘HIV-positive’ identity had been sequestered by the public health logic of TasP; from a public health perspective the chances of onward transmission from her body now equal that of someone without the virus and her personal stewardship of the virus has now diminished as medication replaces her agentic efforts. She explained her partner had questioned the necessity to continue to take ARVs if she was ‘HIV-negative.’ This use of HIV-negative is significant because it suggests the transformation of HIV identity and the part promise of the biological effects of ARVs. It also points to the interpersonal character of these transitions and new questions and conversations on HIV citizenship and responsibilities. This example figures the use of ARVs and specifically TasP as disruptive technology. That is, TasP is a new technology which engenders new types of social relationships, including potential new risks (Webster, 2007).

Taken together, these examples show that TasP identities and related questions of responsibility and interactions with others are deeply intersectional, influenced by individual medical biography, HIV generations, sexual cultures and belief in biomarkers. This intersectional viewpoint guards against monolithic framings of the transitions occurring in how people with HIV and those in their social environments are engaging with the prospects and affordances of TasP. It also indicates considerable policy challenges and attention to diversity in the implementation of TasP. For instance, how should TasP and *responsibility* be framed in cases where people diagnosed late with HIV due to serious structural barriers (including stigma) – which in the UK accounts for over 50% of African men and women – and who struggle to achieve and maintain a suppressed viral load? Although the empowering U = U campaign aims to offer support to individuals claiming a specific *undetectable* identity, HIV policy needs to consider how people living with HIV are not always in a physical or socially safe position to engage with others about TasP and which could exacerbate experiences of isolation and stigma (Thomas, Aggleton, & Anderson, 2010).

## Toxic drugs, toxic bodies: HIV citizenship as a politics of embodied biohazards

Participants spoke of toxicity, both in terms of drug side-effects and the idea of a person living with HIV as a biohazard. These engagements with HIV come close to Petryna’s notion of biological citizenship (Petryna, 2002). Petryna detailed the effects of radiation poisoning and the ways in which Ukrainian citizens negotiated access to and enacted their rights through the framing and management of their ‘toxic’ bodies within state and health institutions. While not an environmental disaster, responsibilities of people living with HIV to live as good biological citizens in relation to ARVs were shaped by similar complexities and contradictions. Squire has argued that discourse on side-effects associated with ARVs is more or less foreclosed in the sanctioned TasP discourse on the HIV pandemic (Squire, 2013). The emphasis on the ‘successful’ patient reinforces the idea that TasP is ideological as patients are encouraged to focus more on the benefits of ARVs and less on the drawbacks. In this light, narratives of toxicity and biohazard may offer one way in which people living with HIV can legitimately articulate some of the troubling aspects of living with HIV in the TasP era.

Toxicity was associated with previous generations of effective ARVs provided to patients into the late 1990s, including dramatic side-effects such as drug-related neuropathy, severe gastrointestinal upset and liver damage. Current generations of ARVs are generally seen to have fewer immediate and serious side effects (Newman et al., 2015). Despite this evidence, interviewees spoke of toxicity in connection with ARVs, particularly those participants who started treatment in the 1990s or knew others that did. One man who was diagnosed in the 1980s and who had resisted HIV treatment for years because of its perceived toxicity was concerned about the serious, long-term effects current ARV treatment has had on people he knew.

I just think people should be aware of the long-term side-effects of HIV medication ‘cause I don’t know whether they’ve all been ironed out relating to what I raised about kidney damage … as things stand you’re still liable to experience your organs being quite hammered by the medications because they’re still highly toxic (Gay man, 60s, on ARVs)

Anxiety over the toxicity of ARVs is often seen as a barrier to effective treatment and even an act of resistance on the part of people with HIV, particularly those seen to be from a generation of people diagnosed with HIV when effective, safe treatments were not available. But we can also see discourse on pharmaceutical toxicity as reflecting contemporary emphasis on personal responsibility for the management of the one’s body (Rose, 2007) and in particular, understanding it as a fragile ecosystem for which the subject is responsible. Newman et al. found similar attitudes in relation to perceived toxicity of ARVs amongst participants living with HIV in their Australian research with many using this as a reason to reject treatment and TasP, consistent with a narrative of self-care (Newman et al., 2015a, 2015b).

Talk of toxicity also emerged in relation to HIV itself, its presence in the body and the potential toxic risk it posed to others. This is a long-standing trope of HIV citizenship, which anchors a form of HIV essentialism (Flowers & Davis, 2013), underpins identity in the TasP era. The idea of HIV toxicity emerged as an important theme, not only in relation to risk of transmission and stigma, but also as rationale for TasP and the initiation of treatment. One participant spoke of beginning to think of TasP as reducing risk and enabling sexual partnerships with HIV-negative people:

…what I’ve heard about it’s that, you’re more infectious when you have a much higher viral load, cause there’s more of it about, and then when people talk about undetectable stuff, that, you know, you’re less infectious obviously when it’s undetectable, and I think, I’ve read something about people are, people think that…you don’t, you can’t pass it on if you’re undetectable…the risk is really really low. So people who are having children and stuff, that they put the person on the drugs or whatever and then they’re able to have a child because they’re on the undetectable, or they’ve got undetectable. So, it makes me feel slightly less sort of like… toxic. Do you know what I mean?

(Gay man, 20s, not on ARVs)

The participant engaged with potential benefits of TasP, summarised for him by the idea of becoming a less toxic embodied self. The extract is exemplary of the ways in which many people in our research engaged with TasP, but it stands out in that it revisits the discourse of HIV as a biohazard. The use of toxic is dense with the implications for identity and related questions of both bodily integrity and, relatedly, biological citizenship. In addition, viewing HIV as toxic opens up the possibilities TasP offers for the reimagining of life with HIV.

The impact of HIV as toxic is made apparent in the ongoing bodily management in serodiscordant environments. One African participant described how, upon recently discovering that she was living with HIV, quickly came to understand how the presence of HIV in her body distinguished her from her partner and children who were HIV-negative.

I remember when I’d just been diagnosed and after he went as well, they were telling him about, you know, being careful with toothbrushes and all that. I kind of really felt, I felt, I don’t know what to say, stigmatised or what but, you know, he came and told me that all they said was we should be careful and all that…sort of like, the way they spoke to him, it was like putting me on that side, and him and the children on the other side. So, what I did, I was like “ok, I’ve got the message”. I went out and bought my toothbrush, electric toothbrush, so that I can stand it on its own, not next to all the others, because I’m thinking ‘oh people are thinking I’m going to transmit through this’, so I, sometimes it gets to the point where I just kind of like accept to say that “he’s different, and I’m on, I’m on another, different side as well’ so there are things that cannot remain the same.

(African woman, 30s, not on ARVs)

This participant appeared unaware of how undetectable viral loads reduced transmission. HIV infection was seen as a division between her and her family and, like a contagious agent, needed to be contained. This approach reflects a long-standing form of engagement with HIV which troubles the prevailing TasP mindset and underlines, as above, the intersectional qualities of life with HIV. This participant’s account indicates a complex interaction of the possibilities and promises of TasP with the lived realities of serodiscordant family life, compounded by being a member of a migrant community. For instance, it is well-documented that social and structural factors – such as poverty, under-employment, lack of childcare and lack of representation on service and policy development – directly affect access to and engagement of migrant communities with health information and health services (Dodds, Mugweni, Phillips, Park, & Young et al., 2018). These factors can also play an important role in shaping experiences of HIV citizenship. The account provided here suggests that a TasP context includes ongoing experiences of HIV-related stigma, where health professionals may also be implicated in sustaining a pre-TasP era logic of the sero-divide. Indeed, we have seen elsewhere how health practitioners can pose significant barriers to HIV information and services (Blondell, Kitter, Griffin, & Durham, 2015). Our research is indicative only, but it does support the view that African participants living with HIV had weak concepts of viral load and HIV transmission, in sharp contrast to the gay men living with HIV who appeared to be better served with knowledge and critical engagements, signalling very different knowledge communities. (Freire, 1970) We argue here *not* that communities of gay men have better HIV literacy than migrant communities, but that histories of community responses to HIV, cultural practices, as well as how intersecting inequalities (e.g. socio-economic, gender, racism) affect diverse communities, will shape how clinical information about the management of HIV will be accessed, understood and incorporated into everyday practice (Chinouya & Davidson, 2003). As such, we suggest that these knowledge communities reflect and contribute to a heterogeneity of HIV citizenship.

## ARVs, TasP and responsible use of resources

Our final theme draws together and builds on the previous exploration of identity and toxicity by considering how participants negotiated TasP as a matter of the good management of resources. The provision of TasP evoked concerns around the cost of treatment and the additional burden on an already stretched publicly funded health system. This is another way in which participants enacted HIV citizenship and locates them in contemporary currents in the political economy of health care, in particular, the transformations of the UK National Health Service (NHS) in an era of scarcity (Keogh, 2017). This also points to the real tensions in how discourses of TasP consider the ‘HIV-positive’ subject in contrast to how economic contexts demands very different types of health citizenship.

One participant described how he had been offered TasP at a regular HIV consultation because he had been repeatedly diagnosed with sexually transmitted infections (STIs). He had explained to his doctors that he did not want to start TasP because he believed the STIs were coming from sexual partners who were living with HIV, with whom he did not use condoms. In contrast to how his clinicians appeared to have interpreted his results – as a sign that he was not using condoms with *any* of his sexual partners and therefore potentially putting HIV-negative partners at risk of HIV – he explained that he always used condoms with HIV-negative partners, and felt that his current strategy of serosorting in relation to condom use was an effective method of HIV prevention. His preference for serosorting over TasP as a method of HIV prevention was coupled with talk of starting treatment as a waste of resources.

I did think about it, yes, but then I thought, well I feel healthy and I’m not really getting a lot of colds etc, so I don’t see what benefit it’d be if I can cost the NHS more to give me treatment then, I could have another couple of years without it and save the NHS a couple of thousand pounds.

(Gay man, 20s, not on ARVs)

What is key here is how the language of cost-effectiveness and savings he could personally help to provide the NHS inflects and supports his decision about TasP and HIV prevention. He and other participants believed ARVs to be expensive, and the potential burden they posed for the health system. The framing of TasP as a prevention strategy at an individual level conflicted with the cost implications of treatment, especially when existing prevention methods – serosorting – were seen to be effective. This approach to treatment decisions sits within wider perceptions of scarcity, not to mention debates about the economic rationality of HIV prevention. Indeed, as we have seen in recent discussions around the cost of pre-exposure prophylaxis in England as a reason to delay its implementation (Boseley, 2016) or decisions about denying treatment to smokers or people who are obese (Campbell, 2016), concerns about who is rightfully entitled to the scarce resources of the NHS becomes much more focused on the *responsible* and socially legitimate citizen. Moreover, this participant’s reading of his healthiness (not getting colds and feeling healthy) is an important reminder of how interpreting bodies is tied to health contexts. Other participants who were not on treatment also spoke of wanting to delay treatment as long as possible and highlighted this as a sign of good health and their *success* as people with HIV. Newman et al., (2015a) have noted that treatment refusal or delay is not necessarily a lack of care for the self but is aligned with caring for the self and a focus on well-being (Newman et al., 2015a). Our research echoes this work and shows how HIV citizenship in the TasP era is articulated with approaches to care of the self.

In contrast, a number of participants described how starting treatment upon diagnosis was something they felt was a responsible act, underlining in another way, the deeply intersectional character of HIV citizenship in the context of TasP. One African woman explained how she needed to do everything she could to ensure her continued good health. This approach to self-care meant starting treatment right away, in spite of the doctor’s advice that it was not entirely necessary at the point of her diagnosis:

I believe the medication really controls, you know, every illness in the ….and for them to tell me “we can start later or we can start now,” instead of it getting worse, it’s better I just start now and see how it’s going to be….because from my perspective of hearing about HIV in my country, was most people died… the medication they were giving them, it just bloated them up, they were getting swollen and like getting, putting on weight, and the families will be fooled like they’re getting better, think the first medication they were being given, and then someone being told “oh he’s looking better;” in a few days time they hear that person has died… So I thought ‘I’m in a better world where I’ll have the better medication and I can, I might as well just start it now.’

(African woman, 30s, on ARVs)

This woman’s account locates her decision to start treatment in her knowledge of previous eras – and geographic locations – of ARVs and their failures. Presented with an expert view that treatment could be taken up or delayed and that either course of action would be OK, she opted to make the leap straightaway. Similarly, one participant explained that there was no question about whether or when he would start treatment:

When I was told about the results they said, “it’s all up to you when you want to start the – taking up the medication.” And then, I just told them any time I’m prepared to take it, take the drugs anytime. So, I took the drugs just when I was told [diagnosed with HIV] yes, I started taking up.

(African man, 60s, on ARVs)

Both examples suggest that starting treatment right away was something which demonstrated responsibility in *doing something*. Although we can only speculate that this decision is informed by an economic rationality concerning the failures of past treatment approaches and the experiences and/or perception of poor ARV provision in African countries, it does point to further complexities of how HIV citizenship and responsible action are tied to questions of national citizenship, health systems and ARV provision. Indeed, TasP brings with it a stark contrast in both global inequities of access to and success of HIV treatment, as well as wider economic inequities between the Global South and the Global North. Thus, in addition to diverse experiences of national citizenship and health systems, these global inequities can play a significant role in relationships with health providers, attitudes to accessing treatment and *responsible* use of resources. While the perspectives on the need to take up treatment right away was not limited to – or even shared by all – African participants living with HIV, the comparison of HIV experiences between the UK and elsewhere played a critical role in how individual responses to treatment initiation were viewed.

## Conclusion

Our research has shown how HIV citizenship forms an integral part of the intimate and everyday lives of people living with HIV in the UK in a TasP era. Responses to TasP in our study disrupt the biomedical narrative which claims a straightforward end to HIV through scaling up access to treatment. Pharmaceutical positioning in relation to treatment, but also cure, biomarkers and social identities illustrate how TasP is already in the process of transforming diverse HIV identities and prevention practices. Toxicity of drugs – and bodies – were seen as something to manage and linked to the shifting possibilities in serodiscordant environment. However, the need to intimately monitor viral loads and related prevention practices have important biopolitical implications for self-management, the increasing molecular surveillance of certain bodies and material inequalities between HIV communities (Petryna, 2002; Rose, 2007). Finally, a sense of being healthy *and* responsible, including appropriate use of resources (NHS-funded treatment), meant conflicting relationships with if and when to start treatment. These findings show how competing notions of responsibility, and enactments of HIV citizenship, are influenced by intersectional and temporal experiences of both sexual practices and health systems.

Where Paparini and Rhodes (2016) described the analytic limits of biological citizenship when health systems and technoscience are barely visible, we suggest that the presence or knowledge of TasP and treatment – rather than the active taking of treatment or *practising* of TasP – plays a critical role in shaping enactments of identity and self-care. Although Paparini and Rhodes questioned the extent to which biological citizenship can operate within resource-scarce settings, citizenship (HIV, biological or therapeutic), is also about identity formation in relation to illness and how HIV is imagined in the TasP era. Indeed, our research highlights how HIV citizenship is not determined by political boundaries but is negotiated and influenced by intersectional experiences of geography, health systems, and ongoing experiences of illness and treatment. We argue that the intimate and everyday *management* and *monitorin*g of the self is a rich space within which to consider HIV citizenship.

This article also adds to the wider literature on illness, risk and biological citizenship as it pertains to ethics of the self, what Rose describes as ‘a set of techniques for managing everyday life in relation to a condition, and in relation to expert knowledge’ (Rose, 2007, p. 146). Active biological citizenship is about acts of risk calculation, choice, and the imperative to take ‘appropriate’ steps to maximise ‘health’. As Rose explains, ‘these enactments of responsible behaviours become routine and expected and are built into public health measures to produce new types of “problematic persons”’ (Rose, 2007, p. 147) and is of particular relevance to our research. Where clinicians may now identify the non-adherent or problematic patient if they refuse or delay treatment, our work highlights how social inequalities, stigmas and understanding of illness (and the limits of cure) itself plays a critical role in how risks to the self are identified and negotiated. This has implications for the introduction and integration of new scientific information about illness and care. In particular, our work shows how active biological citizenship can emerge in multiple forms and through diverse knowledge communities. Routes to self-optimisation may not, in all cases, involve following clinical advice but also, for instance, be about navigating social stigmas, integrating choices about starting medication with wider experiences of health systems, attempting to use resources responsibly and managing intimate familial relations in the face of perceived risks of transmission. The literature on biological citizenship is significant and ever expanding (Happe, Johson, & Levina, 2018). Our research contributes to this field in offering not only nuance in experiences of these forms of self-governance and risk management, but also traces how forms of citizenship may shift over time and in relation to changing scientific understandings of risk.

Our work also points to what Petryna and Follis (2015) describe as risk to citizenship and fault lines of survival. They argue that citizenship as a project is not, nor has ever been, a stable concept but sits uneasily within formal legal, biological and social frames. Where citizenship is an ‘active fault’, sudden repositioning – in our case, a change in scientific understandings of risk of transmission – can result in dramatic shifts, raise hidden or buried histories, and challenge the progressive assumptions that citizenship may lead to improved social conditions (Petryna & Follis, 2015, p. 403). Although our work does not engage with the European migrant ‘crisis’ and ‘bare-life’ as Petryna and Follis explore, it does highlight how diverse communities and experiences of stigma, illness, and access to information and health care on the basis of national identity or migrant status can result not only in widening inequalities but can push some toward the fault lines of citizenship. Axes of identity and social location, such as being part of a migrant community or living with a stigmatised condition, play directly into how rights to health information and services may be constrained. Where Sparke offers the notion of biological sub-citizenship (Sparke, 2017) when exploring how vulnerabilities and lack of rights, we suggest this is not as helpful when it comes to critical engagement with varied forms of self-governance and management of risk. Rather, considering where, how, and when moves towards the fault lines of biological citizenship emerge allow us to better understand what Ann Pollock describes as the ‘multiple forms of biological citizenship’ (Pollock, 2012, p. 7), how they are enacted and to pay attention to and unpack our ‘assumptions about the social location’ of such citizens (Pollock, 2012, p. 144).

Changing narratives in HIV prevention as a result of pharmaceutical-based approaches have considerable repercussions for people living with HIV. The TasP era brings with it increased responsibility to care not only for oneself, but for others, on a political, bodily and even molecular level. Our research demonstrates that we need to consider the multiplicity of HIV citizenship in a TasP era, where we are attentive to intersectional experiences of care, illness and treatment. As pharmaceutical HIV-prevention moves full-steam ahead (Beyrer 2015) we are only just beginning to understand how the implementation of TasP will intimately shape and be shaped by complexities of HIV citizenship.
